# Demonstration of a Filterless, Multi-Point, and Temperature-Independent Fiber Bragg Grating Dynamical Demodulator Using Pulse-Width Modulation

**DOI:** 10.3390/s20205825

**Published:** 2020-10-15

**Authors:** Joao B. Rosolem, Marcio C. Argentato, Fábio R. Bassan, Rivael S. Penze, Claudio Floridia, Artur de A. Silva, Deleon Vasconcelos, Marcelo A. Ramos Junior

**Affiliations:** 1 CPQD Research and Development Center in Telecommunications, Campinas, SP 13086-902, Brazil; marcio.colazza@gmail.com (M.C.A.); fbassan@cpqd.com.br (F.R.B.); rpenze@cpqd.com.br (R.S.P.); floridia@cpqd.com.br (C.F.); arturs@cpqd.com.br (A.d.A.S.); 2 Centrais Elétricas da Paraíba, João Pessoa, PB 58.000-000, Brazil; deleon.vasconcelos@utepasa.com.br (D.V.); marcelo.agra@utepasa.com.br (M.A.R.J.)

**Keywords:** FBG, PWM, engine, high temperature, ECU, FBG demodulator

## Abstract

We demonstrated in this work a filterless, multi-point and temperature-independent FBG (fiber Bragg grating) dynamical demodulator using pulse-width-modulation (PWM). In this approach, the FBG interrogation system is composed of a tunable laser and a demodulator that is designed to detect the wavelength shift of the FBG sensor without any optical filter making it very suitable to be used in harsh environments. In this work, we applied the proposed method that uses the PWM technique for FBG sensors placed in high pressure and high-temperature environments. The proposed method was characterized in the laboratory using an FBG sensor modulated in a frequency of 6 Hz, with a 1 kHz sweeping frequency in the wavelength range from 1527 to 1534 nm. Also, the method was evaluated in a field test in an engine of a thermoelectric power plant.

## 1. Introduction

For practical reasons, the control electronics for many types of power machinery are usually placed inside or close to the operating environment of the engine. For example, the monitoring modules for dynamic pressure, temperature, and knock are placed directly in contact with the engine [1]. The electronic modules that have been developed for monitoring different types of engines (naval, thermoelectric, planes, military, and automotive) with specific emphasis on durability at high-temperature operation [2,3,4].

Monitoring the instantaneous combustion chamber pressure data is required for the closed-loop control of the fuel mass fraction burned in the engines [5,6]. The pressure sensors for this control must be durable and accurate. Using closed-loop control improves engine performance and reduces the emission of pollutants. The feedback system contains an intelligent data analysis system working with an ECU (engine control unit) to precisely dose the fuel quantity in each combustion cycle of each of the engine cylinders. In thermoelectric engines, the dynamical pressure has peaks higher than 250 bar, and the temperature in the combustion chamber is higher than 300 °C. Piezoelectric sensors used to measure the pressure of the combustion chamber currently are not durable when used continuously in high temperatures (>300 °C) [5]. Thus, this application needs a robust and trusty pressure sensor. 

Optical fiber sensors are a good alternative to electronic sensors in many engineering applications due to some intrinsic advantages, such as high temperature and chemical resistance and potential for long-lifetime operation. A fiber Bragg grating (FBG) sensors have flexible characteristics, low cost and are readily available [7]. Examples of FBG sensor applications include structural health monitoring in civil engineering [8,9], electric power systems [10,11], railways and roadways monitoring [12,13], in oil tanks monitoring and as chemical sensors [14,15], in biomechanics and in medicine [16,17]. FBG sensors can be fabricated using optical fibers made of different materials, such as glass [18], polymers [19], or sapphire [20].

Another issue of sensing system operation in a thermoelectric power plant is regarding the FBG interrogators. Although many commercial FBG interrogators modules can measure parameters running in frequencies higher than the engine’s combustion cycles, they cannot be installed close to or inside the engines. The thermoelectric engine is high power machinery that dissipates a lot of heat. The temperature can vary depending on each part of the engine casing. For example, at the pressure monitoring point, the temperature in the case is higher than 300 °C. In the other parts that have water cooling, the temperature is lower. The average temperature in the machine room reaches 55 °C. Considering that the ECU and the FBG demodulator need to be installed close to the engine or its external body, the high temperatures will affect the operation of a supposed interrogation module. In the interrogation module that uses lasers, semiconductor optical amplifiers (SOAs) and Fabry-Perot filters [21], the cooler of the optoelectronics elements of the interrogator, will work in an excessive regime of operation, causing the device to fail in advance [22]. Passive devices such as WDM multiplexers, splitters, circulators also will be affected by the high temperatures. On the other hand, the current technology of electronic devices that can be used in a high-temperature environment is much more available than optoelectronic technology [2]. In addition, standard interrogators are not able to send the signals from all FBG pressure sensors obtained on each sweep to each engine ECU. Many commercial FBG interrogators record the measured data in an external computer in csv or text files. When a single data reading must be taken for control of each cylinder, the receiver of the optical sensing system could use, for example, the optical-edge-filtering technique [23] to detect the dynamic pressure. However, the FBG center wavelength shift due to temperature variation in the combustion chamber depends on the engine load [24]. Thus, the correct positioning of the optical edge filter in real operation is critical to reproduce the exact dynamic pressure behavior of the combustion chamber. Although some techniques were implemented to solve this problem [25], realizing this measurement in a real application is still a serious challenge and the complexity of the optical source and filter control of the edge filtering technique limits its use in this application. An alternative method based on the dispersion delay effect of a dispersion-compensating fiber (DCF) can also be used to convert the FBG wavelengths into the time domain [26], but its demodulator cannot be used near the engine. 

In this work, we proposed an innovative filterless, multi-point, and temperature-independent FBG dynamical demodulator using the pulse-width-modulation (PWM) technique. PWM is a modulation technique that generates variable-width pulses to represent the amplitude of an analog input signal [27]. In [28], an interrogation system is presented based on pulse-modulation, that automatically recognized reflection signals of FBGs even when the FBGs are installed in an arbitrary order or at a long distance and affected by delays. This recognition technique was realized using pulse-modulating in the wavelength-swept laser. However, in contrast to [28], our proposed scheme has a tunable laser that sweeps a pre-set wavelength band where the FBG sensors work in continuous-wave (CW) mode. The demodulator using robust electronic devices can be used near the engine, near the pressure sensor and even integrated with the ECU to transform the wavelength variation-based signal, to a PWD signal and finally in an analog intensity signal compatible with the ECU input port. This approach can be used to measure many types of parameters using FBG sensors. In this work, we applied this method for FBG sensors placed in high pressure and high-temperature environments. The system was characterized in the laboratory using an FBG sensor modulated at a frequency of 6 Hz and a tunable laser with a 1 kHz sweeping frequency and wavelength range from 1527 to 1534 nm. A commercial FBG interrogator was used to compare the results obtained in this application. Besides, it was evaluated in a field test in an engine of a thermoelectric power plant.

## 2. The Proposed FBG-PWM Demodulator

**Figure 1:**
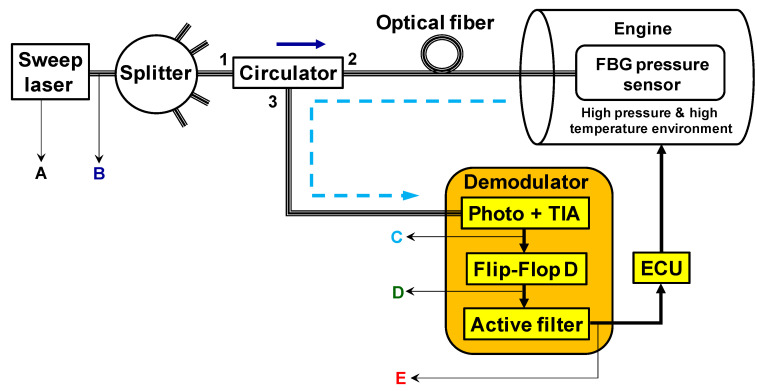
The proposed FBG-PWM demodulator. The letters from A to E represent points with signals waveforms from the sweep laser to the demodulator output.

Figure 1 shows the entire interrogation system where the FBG-PWM demodulator is used. The optical source for this system is a tunable laser with an appropriate sweep frequency. The sweep frequency must be higher than the maximum sensor frequency response to have enough sampled points during the measurements. In the diagram of Figure 1, the laser output can be divided for many demodulators in a power plant. Each splitter output is connected to the sensor using port 2 of an optical circulator. The FBG sensor in Figure 1 is used to measure dynamical pressure inside an engine of a thermoelectric plant engine where the temperature is very high (>400 °C) and is not stable. Port 3 of the circulator is used to connect the FBG reflected signal to the demodulator input. In the demodulator unit, the FBG wavelength-shifted signal follows first to the photodetector. In the photodetector, the optical signal is converted to electrical. Next, a transimpedance amplifier amplifies and clips the signal. Next, a flip-flop type D (FFD) digital circuit transforms it in a PWM signal. Next, low pass active filters are used at the output of the FFD to smooth the pulse train into a stable analog voltage. This analog voltage is the recovered FBG wavelength shifting signal. In other words, the variation of pulse width (PWM) is converted to an analog voltage directed related to the FBG wavelength shifting that in turn, is related to the original engine cycles modulation. This signal is sent to an ECU, which analyzes the signal and provides the correct commands to the engine in closed-loop control.

In this proposed sensing system scheme, only the pressure sensors and the demodulators need to be placed close or on the case of the engine integrated with the ECU. The tunable laser mainly can be installed in a room with controlled temperature.

The key elements to implement the filterless, multi-point, and temperature-independent FBG dynamical demodulator are a tunable ring sweep laser [29] and the FFD digital circuit plus the active filter. The tunable source for this application can be fiber lasers based on semiconductor optical amplifiers or erbium-doped fiber using the Fabry-Perot filter. These lasers have narrow linewidth (<5 pm) and high output power (>10 dBm).

Once the laser output changes in wavelength overtime during the sweep, the optical wavelength variation of FBG in the engine becomes an electrical time variation in the demodulator. When the FFD receives the analog time variation signal of the FBG, it transforms this signal in a digital signal form with a pulse width variation. The pulse width variation has the information of dynamical pressure modulated in the FBG. Low pass active filters (two second-order Butterworth low pass filters) are used at the output of the PWM circuit (D) to smooth the pulse train into a stable analog voltage. The electrical-active-filter removes the digital modulation of the PWM signal recovering the original FBG modulated-signal. Then, this recovered signal is sent to the ECU. No synchronization signal is necessary for this system. 

**Figure 2:**
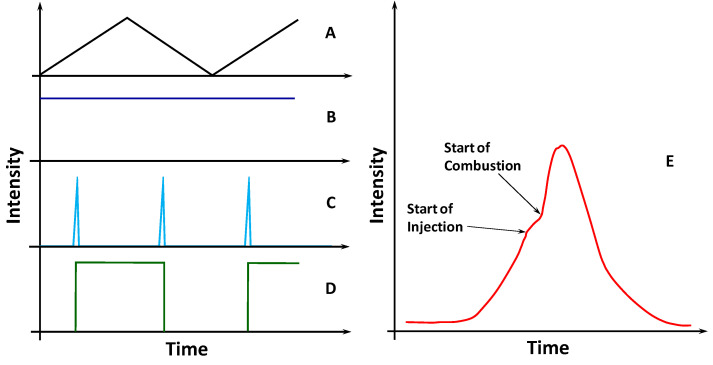
The letters from A to E represent points with signals waveforms from the sweep laser to the demodulator output. In this figure, the signal in E shows a typical engine combustion cycle.

Figure 2 shows the signal waveforms from the tunable laser to the demodulator output. In this figure, the signal E shows a typical engine combustion cycle. The signal-A shows the electrical sweep of the tunable laser. The signal B is the laser output intensity. Although the signal intensity in B is constant in time, the wavelength increases during the positive sweep slope, and it decreases during the negative sweep slope. The signal-C is the FBG electrical signal that is modulated by the dynamical wavelength variation. The signal-D is the digital output of the FFD circuit with pulse width modulation. The signal-E is the active filter output showing an example of a typical engine combustion cycle.

## 3. Demodulator Evaluation in Laboratory

**Figure 3:**
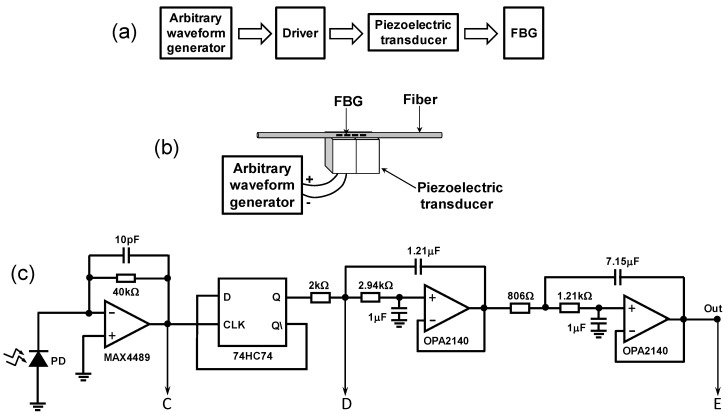
(**a**) Scheme to simulate the temperature changes and dynamical pressures on FBG, (**b**) FBG coupled to a PZT, and (**c**) electronic circuit of the demodulator.

To demonstrate this technique, we first tested the proposed system in the laboratory. Figure 3a shows the scheme to simulate the temperature changes and dynamic pressures on the FBG. An arbitrary waveform generator (BK4054B, B&K Precision Corporation, Yorba Linda, CA, USA) produced a typical 6 Hz engine combustion frequency. This generator waveform voltage was amplified by one piezoelectric driver connected to a piezoelectric transducer (model PK2FQP2- Figure 3b, (Thorlabs, Newton, NJ, USA) that stressed a polyimide coated FBG coupled into the transducer. The tunable laser used a triangle waveform frequency of 1 kHz to sweep the central wavelength from 1527 to 1534 nm. Figure 3c shows more details of the demodulator-electronic-circuit. The photodetector plus transimpedance amplifier has a bandwidth of 400 kHz and, the active filter was composed of two second-order Butterworth low pass filters with 50 Hz bandwidth. The FFD used was a 74HC74 digital circuit.

The type D flip flop circuit changes the output logic level in the rising edge of the signal in the clock input port. The FBG electrical signal from the transimpedance amplifier circuit is inserted into the type D flip-flop clock input port and, the inverted flip-flop output port is connected to the input port of the same chip. Thus, when the swept light is reflected by the FBG just the rising edge of the spectrum alters the flip-flop output. When the sweep occurs from shorter to longer wavelengths (positive slope of signal A), it is the left edge that changes the flip-flop output, and, when the sweep occurs from longer to shorter wavelengths (negative slope of signal A), the right edge changes the flip-flop. Since the FBG is varying dynamically from longer to shorter wavelengths and vice versa, variations in rising and falling edge will create a PWM modulation.

The output voltage in E can be express by (1) [30]:

Vo = δ·V_PWM_ (1)

where V_O_ is the averaged output voltage, δ is the duty cycle of the PWM waveform and V_PWM_ is its amplitude. Considering that the tunable laser sweep time determines the total spectral range (BW) and the FBG produces a dynamical time variation signal proportional to dynamical wavelength shift (Δλ), δ can be written as:

δ = Δλ/BW(2)

and V_O_ can be written as:

Vo = (Δλ/BW)·V_PWM_ (3)

**Figure 4:**
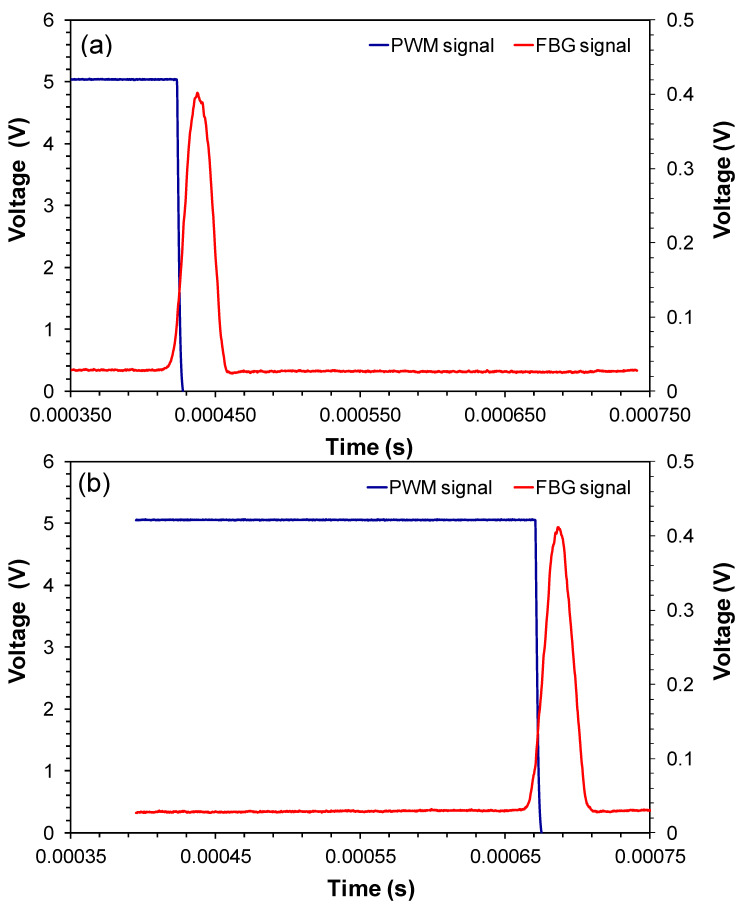
(**a**) and (**b**) Signal in C and D respectively for FBG position in minimum and maximum PZT strain.

**Figure 5:**
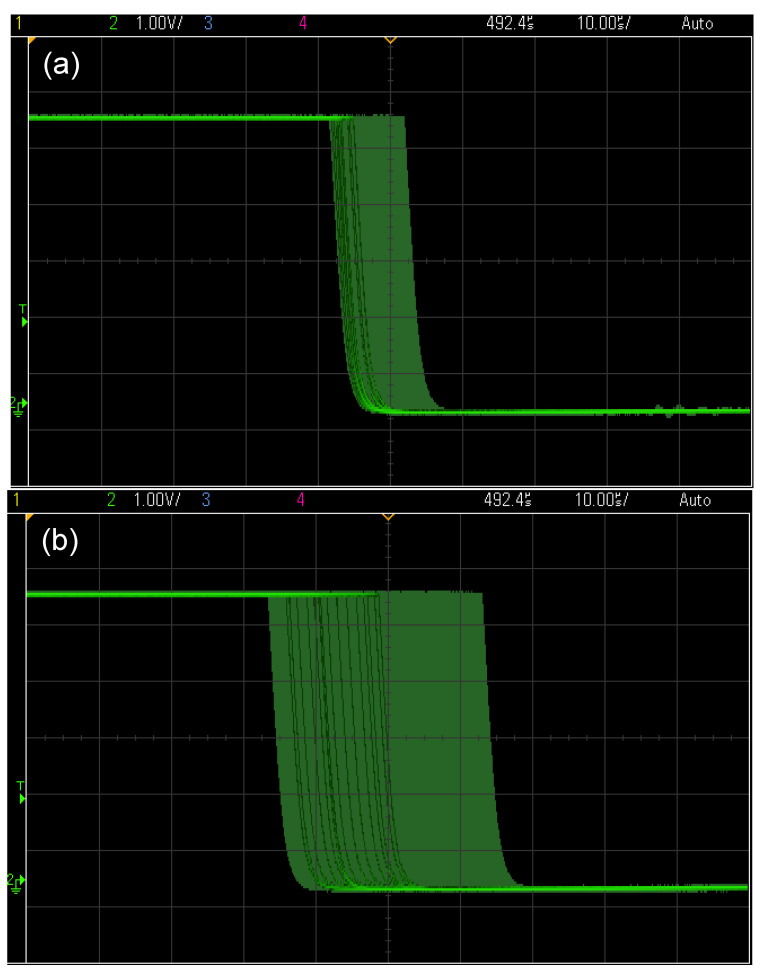
PWM signals in D for two distinct PZT strain amplitudes, (**a**) strain amplitude = 0.2 Vpp and (**b**) strain amplitude = 1.4 Vpp. The vertical axis is voltage (1V/div) and the horizontal axis is time (10 μs/div).

Therefore V_O_ can be increased without reducing the noise-signal ratio reducing the spectral range of the sensing system. Figure 4a,b show the measured signals in C (red) and D (blue) respectively for FBG position in minimum (a) and maximum PZT displacement (b). Figure 5a,b are the PWM signals in D for two distinct PZT displacement amplitudes. These signals were measured in E using an oscilloscope.

**Figure 6:**
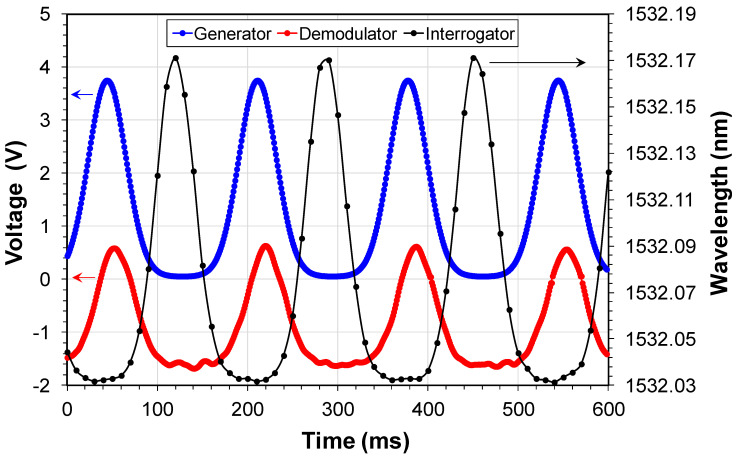
Qualitatively comparison of the signal of the waveform arbitrary generator, the signal in the demodulator output, and the signal measured using a commercial FBG interrogator.

A critical evaluation regarding this proposed system refers to the characteristic of the PWM demodulated signal in terms of trustworthiness to the original FBG modulated signal. We compare in Figure 6 the modulation signal of the arbitrary-waveform-generator, the FBG signal measured in E, and the signal measured of FBG using a commercial FBG interrogator (100 Hz sweep frequency si155 Hyperion from Micron Optics, Atlanta, GA, USA). The commercial interrogator signal was obtained after post-processing. We can observe in Figure 6 that the demodulated signal in E is a good copy of the generator signal waveform; however, some noise can be observed in the signal base. We will comment on the noise source in Section 4. Also, we observed that the signal of the commercial interrogator has not enough sampled points to define all the events in an engine cycle curve. Next, we evaluated quantitatively, the demodulated signal characteristics.

**Figure 7:**
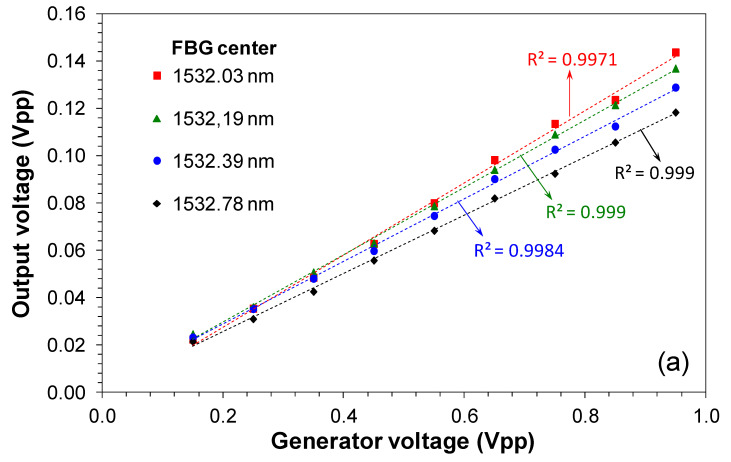

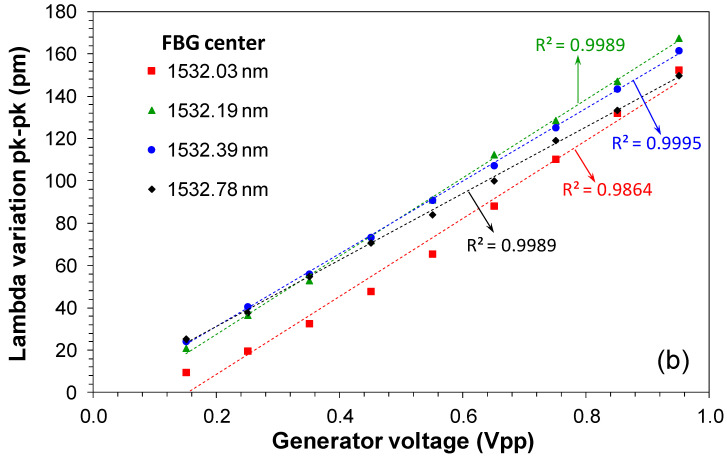
Signal linearity measured in four FBG center wavelengths, (**a**) measured in the demodulator output (E point) and (**b**) measured by a commercial FBG interrogator.

Figure 7a,b show the linearity performance respectively for the demodulator and for the commercial interrogator for four different FBG center wavelengths from 1532.03 to 1532.78 nm. The optical input power in the photodetector was −16 dBm. 

These wavelengths centers represent the FBG at different temperatures inside the engine. This wavelength range (750 pm) corresponds to a temperature variation of 57 °C for an FBG sensitivity of 13 pm/°C [24]. In this measurement procedure, we were limited in the wavelength range due to the PZT voltage limitation. The demodulator could measure the signal in the entire wavelength range determined by the tunable laser sweep (7 nm). We observed that the signals’ linearities in the demodulator output are enough to reproduce the original characteristics of the FBG modulated signal with good quality. We attributed the variation in the offset of the curves to the PZT technical characteristics that are not stable with the time. 

## 4. Demodulator Evaluation in a Field Test

The objective of the field test was to verify the performance of the PWM demodulator in terms of signal processing using a dynamical FBG pressure sensor installed in an environment with variable temperature. It was not the test proposal to test the demodulator itself at high temperatures in this project stage. The field tests take place in Centrais Elétricas da Paraíba (EPASA), which is a thermoelectric power plant. This thermoelectric power plant has an installed power of 340 MW, obtained from 40 model 3240 engines (MAN Diesel SE, Augsburg, Bavaria, Germany). The angular speed of each motor is 720 rpm, and heavy fuel oil (OCB1) is used to combustion engines. Each engine has 18 cylinders and uses a mechanical injection pump to control the fuel oil injection. This mechanism reduces the possibility of adjustments in the injected fuel volume and the same proportion limits the better management of the engines. In the field tests, the pressure sensors were connected in a pressure monitoring point available for each engine cylinder. To compare the pressure signals, we used again the commercial FBG interrogator and the data previously obtained from a reference sensor (model HLV 4.0 from Kistler Group, Winterthur, Switzerland), which is a standard sensor used in the thermoelectric power plant.

The thermoelectric power plant has a harsh environment. The internal average temperature in the machine room is around 55 °C, and close to the engines, it can be higher, limiting the continuous uses of standard electronic equipment.

**Figure 8:**
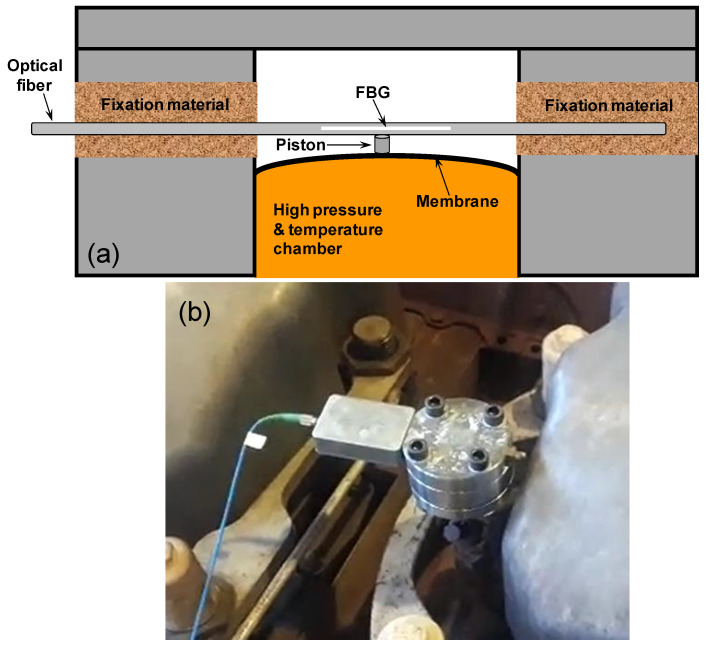
(**a**) Scheme of the FBG pressure sensor and (**b**) sensor connected in the pressure monitoring point of the engine.

Figure 8a shows the pressure sensor scheme [31]. The FBG was fixed in two points of a stainless steel substrate. A pre-stress was applied in FBG before the fixation. According to Figure 8a, the FBG is placed outside of the engine combustion chamber, and it is stressed by a mechanism composed of one 1-mm thickness membrane and one piston. Only one side of the membrane contacts the high-pressure and high-temperature gas inside the engine’s combustion chamber. When the membrane is deformed, by the pressure, it moves a piston that stresses the FBG accordingly. In the field tests, we use FBG pressure sensors connected in a point of pressure monitoring available for each engine cylinder, as we can observe in Figure 8b. 

**Figure 9:**
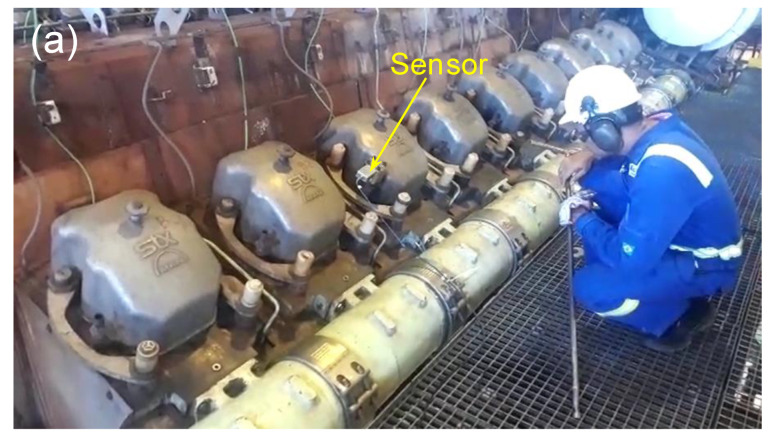

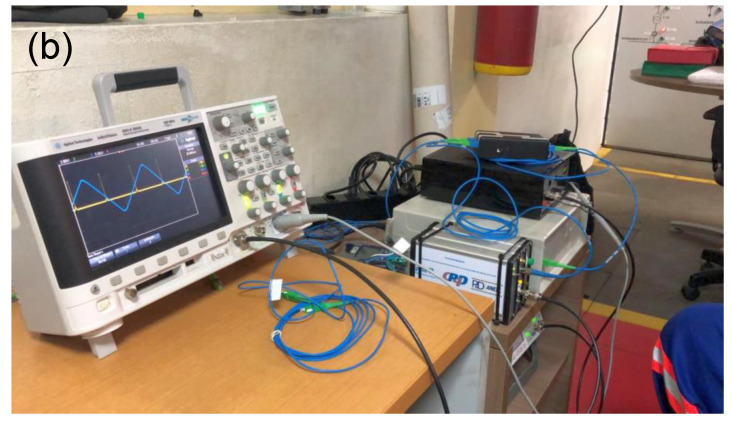
(**a**) Point in the engine where the FBG pressure sensor was installed and (**b**) interrogator and the demodulator tool kit installed in a control room 50 m from the engine under test.

Figure 9a shows the point in the engine where the FBG pressure sensor was installed. Figure 9b shows the demodulator, the tunable laser kit, and the interrogator installed in a control room, 50 m from the engine under test.

**Figure 10:**
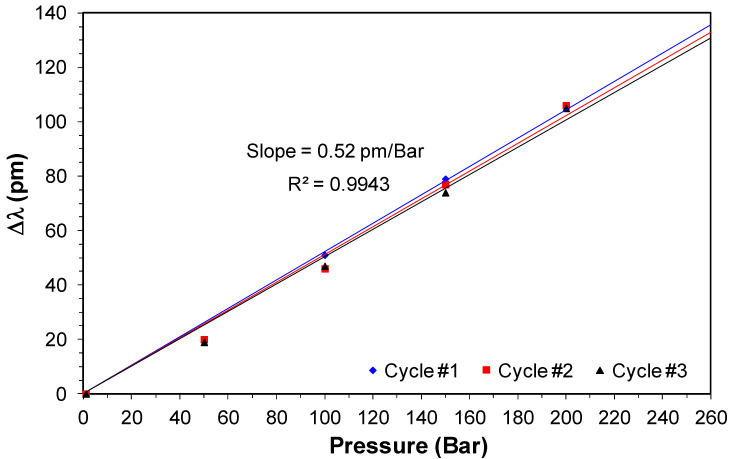
Wavelength shifting versus pressure sensor for sensor #1 used in the field test.

Figure 10 shows the curves of wavelength shifting versus pressure for one FBG sensor obtained previously of the field trial, considering the Bragg wavelength in room temperature that was 1532.90 nm (sensor #1). This sensor was submitted to 3 cycles of static pressure to verify the sensor hysteresis. As we can observe, the sensor curves are linear. The R2 coefficient is 0.9943. The other pressure sensor used in the field test had the Bragg wavelength at room temperature at 1548.12 nm.

**Figure 11:**
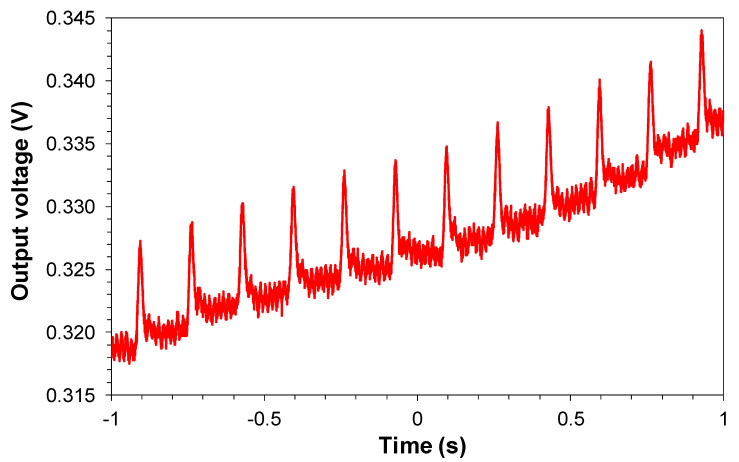
PWM signal versus time of sensor #1 that was obtained during the temperature stabilization period at the engine monitoring point.

Figure 11 shows de PWM signal versus time of sensor #1 at the engine monitoring point obtained during the temperature stabilization period. The Video S1 (in Supplementary Materials) shows the evolution of this signal seen in an oscilloscope during the stabilization period.

**Figure 12:**
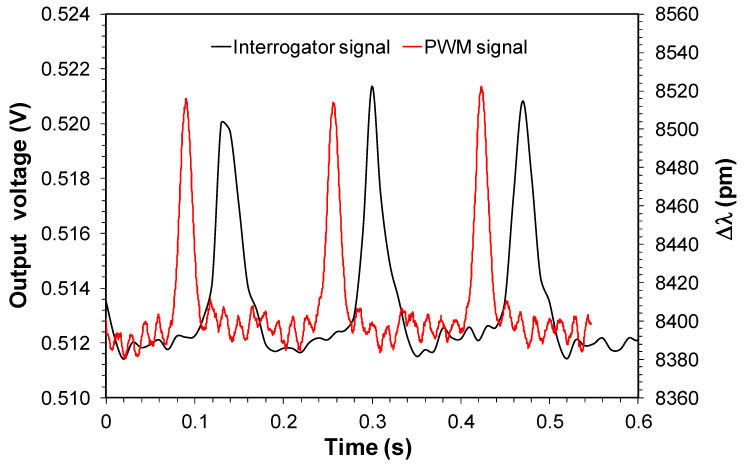
Dynamical curves of FBG pressure sensor in the monitoring point of engine combustion obtained by the PWM demodulator and the interrogator.

Figure 12 shows a qualitative comparison of the dynamic curves of sensor #1 in the monitoring point of engine combustion obtained by the PWM demodulator (with 10 moving average) and the commercial interrogator in terms of wavelength shifting considering the Bragg wavelength in room temperature. 

As we can observe, the output signal intensity of the demodulator in Figure 12 is lower than the one obtained in laboratory measurements showed in Figure 7a. In Figure 7a the peak-to-peak intensity is ~0.12 V versus ~0.009 V in Figure 12. This fact is partly attributed to the higher sweep range used in field tests (~20 nm) compared with the sweep range used in laboratory measurements (7 nm). This higher sweep range was implemented to measure different Bragg wavelengths of two FBG sensors. Also, we observed that the PWM signal presents the narrowest linewidth compared with the interrogator signal. 

The total wavelength shifting showed in the peak bases in Figure 12 for the interrogator signal (~8380 pm) is attributed to FBG sensor substrate deformation and the temperature of FBG in the monitoring point. Based on the stainless steel FBG substrate dimensions where the FBG was fixed (70 mm), its thermal expansion coefficient 16.10^−6^ °C^−1^, and the FBG temperature sensitivity of 13 pm°C^−1^ [24], we can estimate that the temperature operation for this sensor was around 200 °C. 

**Figure 13:**
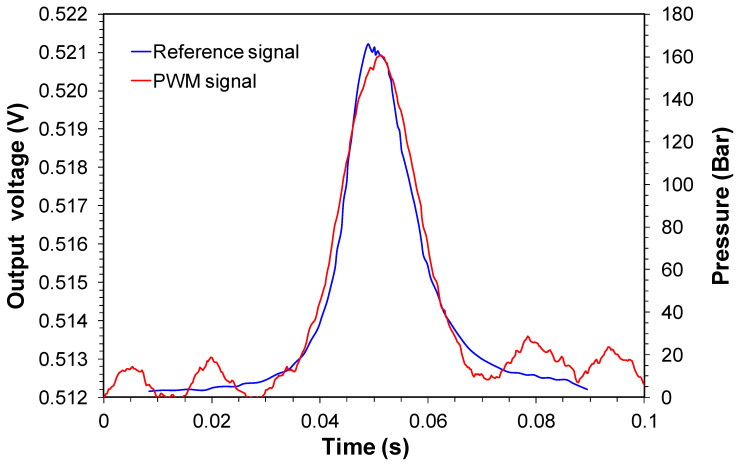
Dynamical curves of FBG pressure sensor in the monitoring point of engine combustion obtained by the PWM demodulator and the reference.

Finally, Figure 13 shows a comparison of the PWM signal and the reference sensor. The time scale was synchronized in order to have a better comparison of the temporal characteristics of the sensors. Except for the noise in the PWM signal base, it shows a response similar qualitatively to the reference sensor. The first hypothesis for noise was due to the tunable laser jitter [32], which would originate from the triangular signal source that sweeps the laser. In our experiments, we used the BK Precision model BK4054B waveform generator. This generator features an RMS 300 ps + 0.05 ppm cycle-to-cycle jitter in 1 kHz and 1 Vpp. Considering the sweep frequency of 1 kHz (1 ms cycle), a variation of 300 ps would have little effect on the creation of the observed noise. A second hypothesis raised would be due to the noise margin in the decision threshold of the Flip-Flop D 74LC74 logic gate, which could widen or shorten the PWM pulses due to the variation of the decision point. This hypothesis was discarded since the voltage levels provided by the optical receiver of the demodulator to the Flip-Flop D inputs were designed to work saturated. Finally, the most likely hypothesis is attributed to the residual noise originated from the demodulator’s power supply. The noise frequency is close to 60 Hz.

## 5. Discussion

To obtain a robustness FBG system for instantaneous combustion chamber control, we demonstrated a filterless, multi-point, and temperature-independent FBG dynamical demodulator using PWM, which can be installed close or inside the engine operating environment.

The demodulator uses just electronic components except for the photodetector. All these devices must be selected for operation in high temperatures. Besides, the demodulator works digitally, which reduces the error on the FBG signal conversion. Because the laser output changes in wavelength and time during the sweep, the optical wavelength variation of FBG in the engine became an electrical time variation in the demodulator. When the demodulator receives the analog time variation signal of FBG, it transforms this signal in a digital signal with a pulse width variation. The pulse width variation has the information of dynamical pressure modulated in the FBG. The active electrical filter removes the digital modulation of the PWM signal recovering the original FBG modulated-signal. This recovered signal is sent to the ECU. No synchronization signal is necessary for this system.

The maximization of the demodulator output signal can be obtained by reducing the laser sweep spectral band to the spectral band of the operation temperature of the sensors. The Bragg wavelength (in room temperature) can be the same wavelength for all sensors in the network, but this condition is not obligatory.

The laboratory tests demonstrated that the proposed demodulator reproduced the engine characteristics adequately. Also, the demodulator signal had more resolution than the commercial interrogator. The laser sweep frequency must be 10 times higher than the maximum sensor frequency response to have enough sampled points during the measurements. We also observed some noise in the signal base (in laboratory and field test) that we attribute to the residual noise originated from the demodulator’s power supply. Also, we measured the linearity of the demodulator signal that is enough to reproduce the original characteristics of the FBG modulated signal. 

In the field test, the demodulator was not tested close to the engine, where we have high-temperatures. The objective of the field test was to verify the performance of the PWM demodulator in terms of signal processing using a dynamical FBG pressure sensor installed in an environment with variable temperature. The design of the electronic-board of the demodulator with high-performance electronic devices is a future project. The demodulator worked well in the field test and, it was proved by the comparison of the PWM signal with the reference pressure sensor.

## 6. Conclusions

In this work, we investigated an innovative filterless, multi-point, and temperature-independent FBG dynamical demodulator using the PWM technique. The demodulator was developed to monitor the instantaneous combustion chamber pressure in closed-loop control of fuel mass fraction burned of the thermoelectric engines. The demodulator must work integrated with an ECU close to the engine, where the temperature is high.

The system was characterized in the laboratory using an FBG sensor modulated in a frequency of 6 Hz and a tunable laser with a 1 kHz sweeping frequency and wavelength range from 1527 to 1534 nm. A commercial FBG interrogator was used to compare the results obtained in this application. Besides, it was evaluated in a field test in an engine of a thermoelectric power plant. The demodulator worked well in the field test and, it was proved by the comparison of the PWM signal with one reference pressure sensor. Briefly, we will evaluate the demodulator, integrated with an ECU, very close to the engine in closed-loop control of fuel mass fraction burned of the thermoelectric engines.

## Supplementary Materials

The following are available online at https://www.mdpi.com/1424-8220/20/20/5825/s1, Video S1: The FBG-PWM signal seen in an oscilloscope during the stabilization period.
